# Design of TiO_2_-Based Hybrid Systems with Multifunctional Properties

**DOI:** 10.3390/molecules28041863

**Published:** 2023-02-16

**Authors:** Simona Ortelli, Maurizio Vespignani, Ilaria Zanoni, Magda Blosi, Claudia Vineis, Andreana Piancastelli, Giovanni Baldi, Valentina Dami, Stefania Albonetti, Anna Luisa Costa

**Affiliations:** 1 CNR-ISSMC (Former ISTEC), National Research Council of Italy-Institute of Science, Technology and Sustainability for Ceramics, Via Granarolo 64, 48018 Faenza, Italy; 2 Department of Industrial Chemistry “Toso Montanari”, Bologna University, Viale Risorgimento 4, 40136 Bologna, Italy; 3 CNR-STIIMA, Institute of Intelligent Industrial Technologies and Systems for Advanced Manufacturing–Italian National Research Council, Corso Pella 16, 13900 Biella, Italy; 4 Ce.Ri.Col, Colorobbia Consulting S.R.L., 50059 Sovigliana-Vinci, Italy

**Keywords:** hybrid system, nano-TiO_2_, lipopeptides, photocatalyst, sorption capacity, antibacterial coating

## Abstract

In recent years, multifunctional inorganic−organic hybrid materials have been widely investigated in order to determine their potential synergetic, antagonist, or independent effects in terms of reactivity. The aim of this study was to design and characterize a new hybrid material by coupling well-known photocatalytic TiO_2_ nanoparticles with a mixture of lipopeptides (LP), to exploit its high binding affinity for metal cations as well as the ability to interact with bacterial membranes and disrupt their integrity. We used both chemical and colloidal synthesis methodologies and investigated how different TiO_2_:LP weight ratios affected colloidal, physicochemical, and functional properties. We discovered a clear breaking point between TiO_2_ and LP single-component trends and identified different ranges of applicability by considering different functional properties such as photocatalytic, heavy metal sorption capacity, and antibacterial properties. At low LP contents, the photocatalytic properties of TiO_2_ are preserved (conversion of organic dye = 99% after 40 min), and the hybrid system can be used in advanced oxidation processes, taking advantage of the additional antimicrobial LP properties. Around the breaking point (TiO_2_:LP 1:1), the hybrid material preserves the high surface area of TiO_2_ (specific surface area around 180 m^2^/g) and demonstrates NOx depletion of up to 100% in 80 min, together with improved adhesion of hybrid antibacterial coating. The last design demonstrated the best results for the concurrent removal of inorganic, organic, and biological pollutants in water/soil remediation applications.

## 1. Introduction

Sustainable wastewater management has become the primary agenda of the sustainable development goals worldwide [1].

This challenge is addressed by technologies that promote a concurrent removal of organic (molecules, oils, microorganisms, etc.) and metal pollutants from contaminated sites (water or soil).

Nano-TiO_2_ offers low cost, high reactivity, and easy recovery of photocatalytic technology. Its light-activated capacity to oxidate/mineralize organic compounds is of great relevance and significance in advanced oxidation processes for water and air depollution [2,3,4,5]. The possibility of activation in the visible light region as a result of metal and nonmetal doping and the fabrication of composites has recently attracted increasing attention due to possible applications under outdoor solar or indoor LED-visible sources of irradiation. The design of TiO_2_ nanoparticles (NPs) strongly affects their efficiency. It is influenced by:(1) quantum size effects such as band gap energy and the light-induced charge transfer between the adsorbate molecules and the substrate; (2) surface area effects such as the light absorption efficiency and the consequent surface photocarrier concentration; and (3) carrier diffusion effects such as the recombination rate of photogenerated carriers.

Lipopeptides are a class of biosurfactants that have been widely studied and utilized for various biomedical and environmental applications due to their diverse properties, including antimicrobial, antiadhesive, antitumor, and bioremediation activities [6,7,8,9,10,11,12]. Lipopeptides possess surfactant properties due to their amphiphilic nature, having both hydrophilic (peptide) and hydrophobic (lipid) components. This allows them to interact with cell membranes, disrupting their structures and functions. As a result, lipopeptides can exhibit potent antimicrobial effects against a wide range of pathogens, including bacteria, fungi, and even some viruses [13,14].

Lipopeptides also exhibits good stabilizing properties used in the sol–gel synthesis of metal nanoparticles [15,16,17,18,19]. Thus, we decided to exploit the coupling between TiO_2_ NPs and a mixture of lipopeptides (LP), to investigate the physicochemical identity of the hybrid phase and the possible synergetic, antagonist, or independent effects in terms of functionality [20,21].

**Scheme 1:**
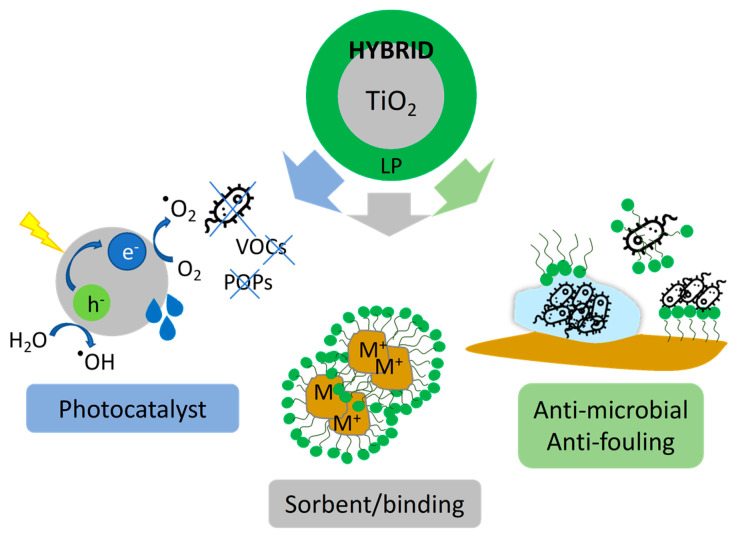
Multifunctional platform designed for the removal of water/soil pollutants.

New inorganic−organic multicomponent materials based on TiO_2_ and LP were produced, characterized, and tested for their multifunction activity, as reported in Scheme 1.

Two synthesis design strategies, namely chemical sol–gel synthesis of TiO_2_ nanoparticles (NPs) nucleated over LP solution and colloidal heterocoagulation, exploiting the attraction between opposite charge TiO_2_ NPs and LP, were used to produce hybrid materials made by TiO_2_.

We tested the photocatalytic activity vs. the degradation of rhodamine B (RhB) in water, the abatement of NO_x_ at gas phase, the sorption of Cu^2+^ ions, selected as probe metal, and the biocidal action following the ASTM E2149 procedure against Gram-positive bacteria *Staphylococcus aureus*.

## 2. Results and Discussion

### 2.1. Sol–Gel Synthesis (TiO_2_@LP_S)

**Figure 1:**
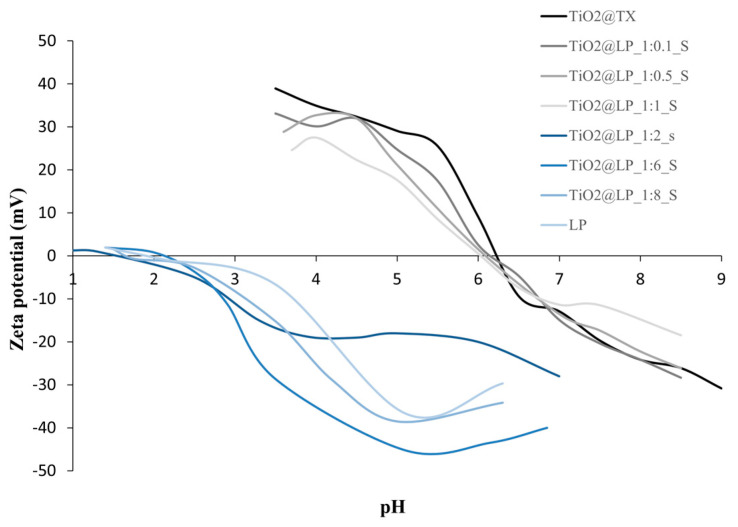
Zeta potential as a function of pH curves for TiO_2_@LP_S samples obtained via sol–gel synthesis.

Data from colloidal characterization of TiO_2_@LP_S samples, differing for the TiO_2_:LP weight ratio (details in Section 3.2.1), are reported in Table 1. We observed an increase in hydrodynamic diameters, determined by DLS analysis (Figure S1 and Table 1), as a function of the LP amount. This was justified by the increase in the steric hindrance of the LP shell and by the destabilization of colloidal dispersion, which were caused by free, non-adsorbing molecules in solution (depletion flocculation) [22]. The zeta-potential curves as a function of pH, reported in Figure 1, show an abrupt change in colloidal properties, passing from a 1:1 to 1:2 TiO_2_:LP ratio. Two populations are clustered around the curves of the two separate components: one with nanometric hydrodynamic diameters, positive zeta potential, and an isoelectric point comparable with that of the TiO_2_ reference sample (TiO_2_@TX) and the other with micrometric hydrodynamic diameters, negative zeta potential, and an isoelectric point similar that of LP. This result offered a clear indication of which design option to select if we wish to prioritize the TiO_2_ or the LP colloidal identity in the hybrid system. 

**Figure 2:**
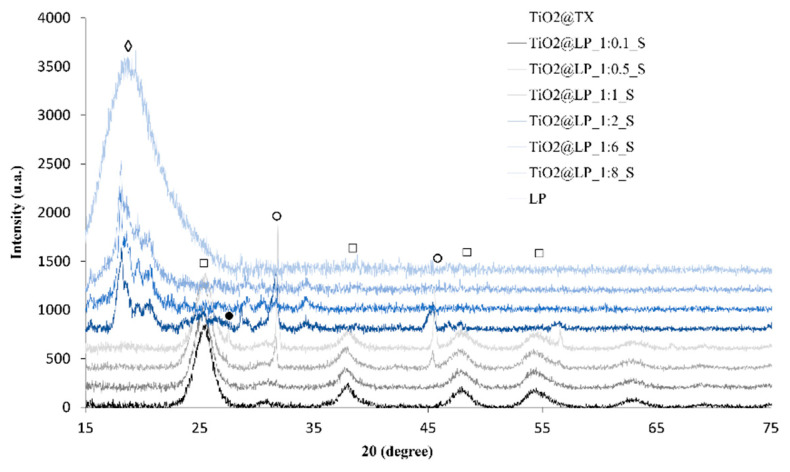
XRD diffractograms of TiO_2_@LP_S_SFD samples (◊, LP; □, anatase; ●, brookite; ○, sodium chloride).

A breaking point, passing from the 1: 1 and 1: 2 TiO_2_:LP ratios, was also observed in the XRD diffractograms (Figure 2). The peaks of TiO_2_ anatase with small traces of brookite were observed up to the TiO_2_:LP 1:1 weight ratio. In the group of samples with higher LP content, the characteristic peaks of the anatase phase disappeared. In this case, we can hypothesize the formation of very small clusters where the ionic phase is still in equilibrium with the solid [23,24] or the presence of amorphous titania phase [25]. In this group of samples, in relation to the high content of LP, we noticed the formation of NaCl as a by-product of the synthesis. This was identifiable based on peaks at 2*θ* = 32° and 47°. At 2*θ* = 18°, a broad peak, which was attributable to the organic phase, confirmed the presence of the high concentration of LP.

**Figure 3:**
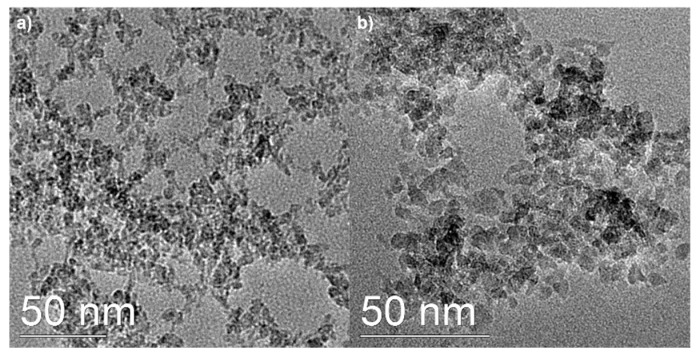
TEM images of (**a**) TiO_2_@LP_S_1:0.1 and (**b**) TiO_2_@LP_S_1:1 samples.

In order to verify the formation of TiO_2_ NPs, TEM analysis was carried out. Figure 3 reports the TEM images of TiO_2_@LP_S_1:0.1 and TiO_2_@LP_S_1:1 sample, confirming the presence of crystalline well-dispersed TiO_2_ NPs, with sizes ranging from 3 to 12 nm and from 2 to 10 nm, respectively. The selected area electron diffraction (SAED) pattern and the relative rotational average (Figure S2) showed that all rings can be indexed as a mixture of anatase (majority phase) and brookite nanocrystals, confirming the data obtained via XRD analysis (Figure 2). By increasing the content of LP in TiO_2_@LP_S_1:2 and TiO_2_@LP_S_1:6 samples, no evidence of the presence of TiO_2_ NPs could be found via TEM analysis. Moreover, no evidence of the presence of crystalline NPs could be detected by SAED patterns. The TEM results confirm the hypothesis that very small clusters in equilibrium with their ionic phase should form at a high LP concentration, as the presence of LP hindered the growth of the as-formed nuclei. 

### 2.2. Heterocoagulation Process (TiO_2_/LP_E)

The physical mixing of oppositely charged TiO_2_@TX_S and LP (Table 2) provided the heterocoagulated samples TiO_2_/LP_E, whose components are mainly bound by electrostatic attractive interactions [26]. The samples were prepared at different TiO_2_:LP weight ratios (see Section 3.2.2 for details). Additionally, in this case, we investigated the colloidal behavior of DLS and zeta-potential measurements and noted an increase in the hydrodynamic size of the multicomponent system with the increase in LP content. In particular, the sample corresponding to the TiO_2_:LP 1:1 weight ratio was the first to reverse the positive zeta potential of TiO_2_, which had dramatic consequences for the colloidal stability (DLS size around 1 μm), as reported in Table 2 and Figure S3a. With higher content of LP, the hydrodynamic diameter decreased, and the negative zeta potential of the composite increased due to the electrosteric contribution of S which, in the case of heterocoagulated samples, acts as a dispersing agent. As was the case for sol–gel samples, the isoelectric points (pH_iep_) of the heterocoagulated samples at high content of LP (1:6 and 1:8 TiO_2_:LP weight ratio) are coincident to that of LP (Figure S3b) even if the breaking point is not so evident, as in the previous case.

**Figure 4:**
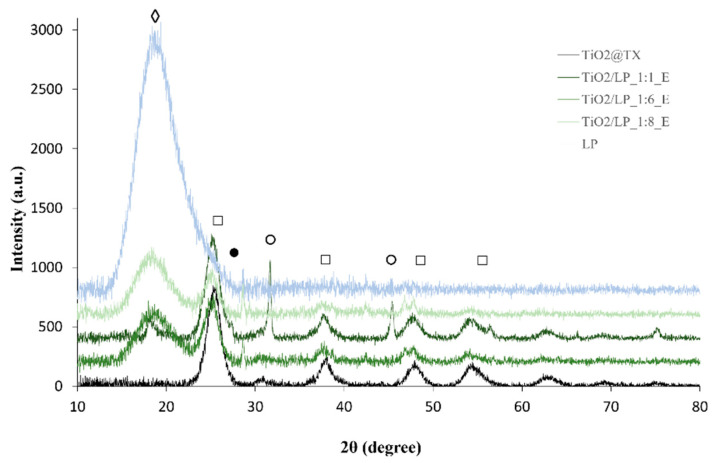
XRD diffractograms of TiO_2_/LP_E_SFD samples (◊, LP; □, anatase; ●, brookite; ○, sodium chloride).

The XRD diffractograms showed the presence of both phases, as expected (Figure 4). TiO_2_ (anatase phase) was recognized from characteristic peaks at 2*θ* = 25°, 38, 48, and 54°. Additionally, small traces of brookite were detected at 2*θ* = 26°. The LP organic phase showed a broad peak at 2*θ* = 18°. We observed a proportional increase in intensity of the LP peak as the LP content in the samples increased; this passed from TiO_2_/LP_1:1_E to TiO_2_/LP_1:8_E. The formation of a synthesis by-product, NaCl, was also identifiable, with peaks at 2*θ* = 32° and 47°.

### 2.3. Comparison between TiO_2_@LP_1:1_S and TiO_2_/LP_1:1_E

The TiO_2_@LP_1:1_S and TiO_2_/LP_1:1_E samples are the most representative for both synthesis methods of hybrid systems; in fact, they represent the breaking point, with a shift of properties from TiO_2_ to the LP component. Therefore, these samples were further investigated by additional characterization techniques (FTIR spectroscopy and BET analysis).

**Figure 5:**
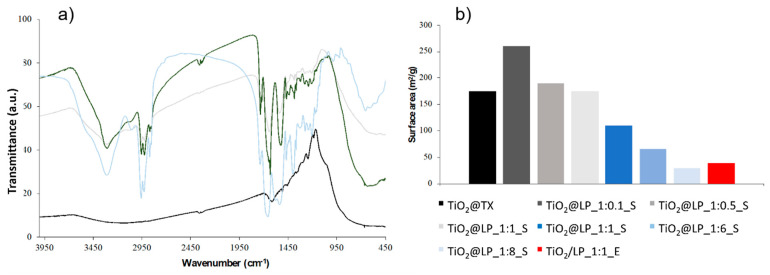
(**a**) FTIR spectra of TiO_2_@TX (black), LP (light blue), TiO_2_@LP_1:1_S (light gray), and TiO_2_/LP_1:1_E (dark green) samples and (**b**) specific surface area data (m^2^/g).

In Figure 5a, comparison between the FTIR spectra of TiO_2_@LP_1:1_S and TiO_2_/LP_1:1_E samples with single components (TiO_2_@TX and LP) is reported. The reference TiO_2_@TX (black curve) showed a broad band at 500–800 cm^−1^ corresponding to the vibration of Ti-O-O bond [27] and Ti-O stretching [28], confirming the presence of TiO_2_. Moreover, we observed some peaks in the wavenumber range 1100–1600 cm^−1^; these are attributable to Triton X. Their low intensity is due to the high TiO_2_:Triton X weight ratio (Table S1). The peaks at 1100 and 1243 cm^−1^ correspond to the asymmetric stretch of aromatic ether; those in the range 1360–1450 cm^−1^ are related to the C-H bending vibration; and peaks at 1511 and 1610 cm^−1^ correspond to the stretching vibration of the benzene group [29]. The light-blue curve in Figure 5a shows the FTIR spectrum of the LP component, which clearly highlights the typical peaks of lipopeptide, with peaks at 3350 and 1528 cm^−1^ attributed to NH-stretching mode and peaks at 2956–2870 cm^−1^ and 1467–1368 cm^−1^ representative of the C–H group of aliphatic chains (–CH_3_; –CH_2_–) with symmetric stretching at 2870 cm^−1^.

The FTIR analysis on hybrid TiO_2_@LP_1:1_S and TiO_2_/LP_1:1_E samples allowed us to confirm the presence of typical functional groups of both TiO_2_ and LP. The main characteristic peaks of TiO_2_ and LP were observed in both TiO_2_@LP_1:1_S and TiO_2_/LP_1:1_E samples (Figure 5a). Through detailed comparison of the two samples, we can observe a lower intensity of peaks corresponding to the LP and the absence of characteristic bands of LP at high wavenumber in the TiO_2_@LP_1:1_S sample (blue curve). On the other hand, in the TiO_2_/LP_1:1_E sample (dark-green curve), obtained by heterocoagulation, all characteristic peaks of LP were recognized. These observations could be justified by a stronger interaction between TiO_2_ NPs and LP in the sample obtained by sol–gel synthesis method, with the formation of a new hybrid phase slightly different from the single component. 

The specific surface areas (LPAs) of spray-freeze-dried (SFD) powders are reported in Figure 5b. Samples synthesized using a sol–gel method at a low LP concentration show high values of surface area comparable with that of the TiO_2_ component. The LPA of TiO_2_:LP_1:1_S sample is five-fold higher than the corresponding heterocoagulated sample. This result is most likely due to intimate molecular interaction between the two components in the sol–gel-synthesized samples, which promotes a real dispersing action of LP over TiO_2_ NPs. The abrupt decrease in surface area that occurs as a result of the increasing TiO_2_:LP weight ratio is in agreement with the increased agglomeration caused by the LP depletion action discussed previously (Table 1). Additionally, the low surface area of the heterocoagulated sample is justified by the high degree of agglomeration of this sample, which decreases the number of TiO_2_-accessible surface sites. 

### 2.4. Functional Characterization

#### 2.4.1. Photocatalytic Tests

The results of photocatalytic tests performed in water and expressed as a percentage of conversion of the rhodamine B (RhB) under visible solar light are reported in Figure S4 and Table 3.

Firstly, no samples demonstrated adsorption of RhB after one hour of contact in the dark (Figure S4a). The TiO_2_@TX reference component had the highest photocatalytic performance, with a conversion of 99% and the highest kinetic constant (9.5 × 10^−2^ min^−1^). The hybrid systems for low LP content showed very good photocatalytic performance, with conversion around 90% and a high kinetic constant (k) up to the sample with TiO_2_:LP 1:0.5. Increasing the content of LP led to an abrupt decrease in photocatalytic activity up to samples TiO_2_@LP_1:6_S and TiO_2_@LP_1:8_S, which did not demonstrate any significant photocatalytic activity despite the photoactivation (absorption in the range between 300–400 nm, reported in Figure S8 and Table S3). The reduction in photocatalytic activity around TiO_2_:LP 1:0.5 and 1:1 weight ratios is in agreement with the abrupt change in physicochemical properties and the previously noted absence of TiO_2_ crystalline peaks detectable through XRD (Figure 2) or TEM SAED analysis. Moreover, the trend caused by the increased amount of LP surrounding TiO_2_ NPs can be justified by the shielding effect of organic LP coating, which depresses the photocatalytic activity of TiO_2_ NPs [30]. 

The results of tests performed using samples prepared via heterocoagulation (TiO_2_/LP_E) are reported in Figure S5 and Table 4.

For samples obtained via heterocoagulation, almost no sorbent capacity was observed for RhB after one hour of contact in darkness (Figure S5a). The samples obtained via heterocoagulation had very poor photocatalytic performance, with conversion data of less than 20% and a very low kinetic constant. As expected, the presence of the LP layer surrounding TiO_2_ NPs, which depresses the specific surface area (Figure 5b), also has a strong shielding effect on the TiO_2_ photocatalytic performances, with a reasonable quenching of radicals produced by TiO_2_ NPs due to their organic coating [31].

Overall, the comparison of photocatalytic performances of TiO_2_@LP_1:1_S and TiO_2_/LP_1:1_E samples highlights how, in the sol–gel-synthesized hybrids specifically, it is possible to take advantage of the highly surface-area-dependent photocatalytic properties of TiO_2_.

#### 2.4.2. Sorption Tests

The results of sorption tests performed on representative samples are reported in Table 5. They are expressed as the Cu^2+^ sorption capacity, which allows it to simulate these materials’ use for the removal of heavy metals from wastewater. The data show similar trends for both sol–gel synthesis and heterocoagulation samples, with a sorption capacity that is dependent on the LP content. Below the TiO_2_:LP 1:1 weight ratio, the samples show the same sorption capacity of TiO_2_, whilst at the highest concentration of LP, the samples show the same behavior of LP alone. This result suggests the main sorption/complexing role of LP is solely dependent on the amount of LP available and not its degree of agglomeration or interaction with the TiO_2_ component. Moreover, we observed no significant increase in Cu^2+^ sorption over time, demonstrating that the sorption ability of all these samples is characterized by fast kinetics.

#### 2.4.3. Antibacterial Tests

Biocidal action was tested on TiO_2_@LP_S nanosols obtained via sol–gel synthesis and de-posited on polyester textile substrates via a dip-pad curing method [32]. In Table 6, the add-on (%) and the bacterial reduction (%) data are reported.

The LP alone showed a low bacterial reduction (40%) due to the low add-on value (1.7%). The presence of TiO_2_ increases the amount of material that can be transferred on the sample, with consequent improvement of antibacterial properties (up to 89% for the TiO_2_@LP_1:0.1_S sample). We also we observed a decrease in performance at the highest LP content despite the highest add-on, confirming that the LPA and availability of free surface sites also play a huge role in the biological reactivity in this case. Overall, it was difficult to determine if synergic effects occurred between the two potentially active components; the only evident effect is the improved adhesion (add-on value) provided by the TiO_2_ phase.

#### 2.4.4. NO_x_ Abatement Tests

**Figure 6:**
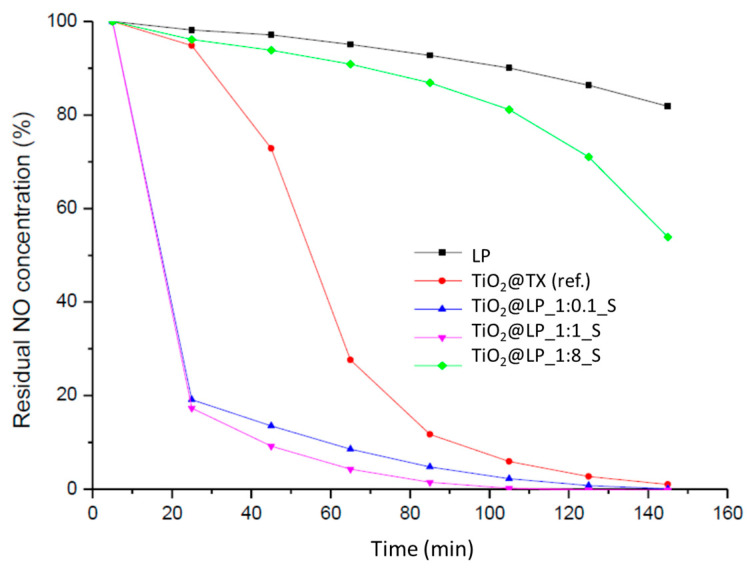
NO depletion trend as a function of UV light time of irradiation.

Photocatalytic performances were also evaluated on TiO_2_@LP_S coated textiles through NO_x_ abatement tests. The data expressed as NO depletion trend as a function of time are reported in Figure 6.

As expected, the inactivity of LP alone was demonstrated by the negligible depletion of NO (LP—black curve). In agreement with this result, low reactivity was also found at the highest LP concentration (TiO_2_@LP_S_1:8—green curve) despite the highest amount of composite deposited on the textile (add-on value reported in Table 6). On the other hand, the reference (TiO_2_@TX) and the hybrid samples with low LP content showed complete NO depletion after 2 h of UV light exposure. This trend concurs with the results of photocatalytic and antibacterial tests reported in Section 2.4.1 and Section 2.4.3, respectively. As expected, at a high concentration of TiO_2_, the photocatalytic properties of the inorganic photocatalyst prevail, with consequent high NO depletion. The higher degradation kinetics of hybrid samples compared with reference TiO_2_ (depletion around 80% for TiO_2_:LP_1:0.1 and 1:1_and <5% for TiO_2_@TX, at 20 min) is highly encouraging and can be justified by the dispersing capability of LP, which maximizes the availability of TiO_2_ active surface sites. (See Supplementary Materials)

## 3. Materials and Methods

### 3.1. Materials

Titanium(IV) isopropoxide, hydrochloric acid, Triton X, rhodamine B and copper chloride were purchased by Merck Life Science S.r.l. (Milano, Italy). The mixture of lipopeptides, was provided as crude extract by AmbrosiaLab (Ferrara, Italy). All the reagents were used without further purification.

### 3.2. Methods

The hybrid materials based on TiO_2_ and mixture of lipopeptides (LP) were prepared using two synthesis design strategies: sol–gel synthesis and heterocoagulation.

#### 3.2.1. Sol–Gel Synthesis (TiO_2_@LP_S)

TiO_2_ nanoparticles nucleated over LP solution were obtained via classical sol–gel synthesis via acidic catalysis starting from titanium(IV) isopropoxide, replacing the chemical surfactant (Triton X) at a very low concentration in the synthesis process (TiO_2_:Triton X 1:0.06-TiO_2_@TX_S) with the mixture of lipopepetides LP [33]. The resulting hybrid TiO_2_ phases (TiO_2_@LP_S) were prepared with varying TiO_2_:LP weight ratios (1:0.1; 1:0.5; 1:1; 1:2; 1:6 and 1:8). The sol–gel-synthesis processes are schematized in Figure S6a. All the samples prepared by sol–gel-synthesis process are summarized in Table S1.

#### 3.2.2. Heterocoagulation Process (TiO_2_/LP_E)

The product TiO_2_@TX_S was mixed with aqueous solution of LP in a pH range in which both surfaces TiO_2_@TX and LP had opposite charges to promote the electrostatic interaction. The heterocoagulated samples were prepared with varying TiO_2_:LP weight ratios (1:1; 1:6, and 1:8) and maintained under stirring for 24 h to favor electrostatic interaction. The heterocoagulation process is schematized in Figure S6b. All the samples prepared via heterocoagulation are listed in Table S2.

#### 3.2.3. Spray-Freeze-Drying Technique 

The spray-freeze-drying technique (Figure S7) was employed here to obtain handy powders (SFD), starting from the TiO_2_- and LP-based nanosuspensions prepared by both methods (sol–gel-synthesis and heterocoagulation process) by means of a lab-scale granulator instrument, LS-2 (PowderPro, Mölndal, Sweden). The nanosuspensions were atomized through a peristaltic pump, blowing nitrogen gas at 0.4 bar through a 100 μm nozzle, and nebulized into a stirred solution of liquid nitrogen to enable instantaneous freezing of each generated drop. The so-frozen drops were placed into a freeze-drying apparatus with a pressure of 0.15 mbar and a temperature of −1 °C to promote the sublimation process, which was completed within 48 h, and a highly porous granulated powder was produced. All the samples prepared via the SFD process are summarized in Tables S1 and S2.

### 3.3. Physicochemical Characterizations

#### 3.3.1. Colloidal Characterization 

The particle size distribution and zeta potential were determined at 25 °C using a Zetasizer Nanoseries (Malvern Instruments, Malvern, UK) via dynamic light scattering (DLS) and electrophoretic light scattering (ELS) techniques, respectively. The DLS technique can calculate the hydrodynamic diameter of suspended particles, and the Smoluchowski equation was applied to convert the electrophoretic mobility to zeta potential. After a 2 min temperature equilibration step, samples underwent three measurements; the hydrodynamic diameter and zeta potential were obtained by averaging these measurements. The instrument is equipped with an auto-titration unit, which enables the identification of the isoelectric point (pH_IEP_) and automatically adds to the sample KOH 0.1 M or HCl 0.1 M in order to explore the zeta-potential trend within a selected pH range. The measurements were performed on the TiO_2_- and LP-based nanosuspensions prepared via both methods (sol–gel synthesis and heterocoagulation) at 0.1 g L^−1^ concentration. 

#### 3.3.2. X-ray Diffraction (XRD)

XRD measurements were carried out on sample powders obtained using a spray-freeze-drying technique (SFD) at room temperature with a Bragg/Brentano diffractometer (X’pertPro PANalytical, Malvern Panalytical, Malvern, UK) equipped with a fast X’Celerator detector, using a Cu anode as the X-ray source (Kα, λ = 1.5418 Å). Diffractograms were recorded in the range 10−80° 2*θ* counting for 0.2 s every 0.05° 2*θ* step.

#### 3.3.3. Transmission Electron Microscopy (TEM)

The transmission electron analyses on TiO_2_@LP_S nanosols obtained via sol–gel synthesis were performed with a FEI TECNAI F20 microscope (FEI F20, Thermo Fisher Scientific, Waltham, MA, USA) operating at 200 keV. The instrument is also equipped with a dispersion micro-analysis of energy (EDS) (EDAX, Thermo Fisher Scientific, Waltham, MA, USA) and the scanning transmission electron microscopy (STEM) accessory (FEI F20, Thermo Fisher Scientific, Waltham, MA, USA). The TEM images were taken in the phase contrast mode and selected area electron diffraction (SAED). STEM pictures were recorded using a high-angle annular dark field (HAADF) detector: in this imaging mode, the intensity I is proportional to Z^1.7^t, where Z is the mean atomic number, and t is the thickness of the specimen. After 1:1000 dilution in water, the nanosols were sonicated for four minutes, then deposited on a holey carbon film supported by a gold grid, and dried at 100 °C.

#### 3.3.4. Fourier Transform Infrared (FTIR) Spectroscopy

FTIR analysis was performed on the TiO_2_@LP samples after the SFD process. The powders were pelletized with KBr, adding ca. 1.25 mg of powder to 100 mg of KBr. The measurements were obtained using a Nicolet iS5 spectrometer (Thermo Fisher Scientific Inc., Waltham, MA, USA). A wave number range between 400 and 4000 cm^−1^ was set for the analysis, and 24 runs were performed for each measurement with a resolution of 1 cm^−1^ using the IR accessory (model iD1). The positions of the peaks were identified by means of the OMNIC software (v9.2, Thermo Fisher Scientific, Waltham, MA, USA) and comparing values with the data in the literature.

#### 3.3.5. Specific Surface Area by BET Method

Specific surface areas of spray-freeze-dried powders were measured by N_2_ physisorption apparatus (Surfer Thermo Scientific) via Brunauer–Emmett–Teller (BET) (Surfer, Thermo Fisher Scientific, Waltham, MA, USA) analysis, in which samples were pre-treated under a vacuum at 200 °C for 1 h.

### 3.4. Functional Characterizations

#### 3.4.1. Photocatalytic Degradation of Rhodamine B (RhB)

Photocatalytic degradation of RhB was conducted in a beaker at room temperature. The typical setup foresees the addition of samples, both in suspension and in powder (at 0.1 g L^−1^ concentration), to 150 mL of a RhB aqueous solution. In order to establish an absorption/desorption equilibrium between catalyst and RhB, the solution was kept in the dark for about 60 min, which proved to be a suitable time to ensure the equilibrium. Absorption/desorption phenomena occurring during the stirring were verified and evaluated as negligible in relation to the overall photocatalytic reaction. The suspension was stirred and irradiated via a solar simulator (SUN 2000 11000 model, Abet Technologies, Milford, CT, USA) with 1000 W intensity. The lamp was switched on before the beginning of the photocatalytic test to stabilize the emission power. The analyses were performed using a quartz cuvette as a sample-holder. The degradation reaction progress was monitored at regular times (10, 20, 30, 40, 50, and 60 min) by withdrawing and centrifuging (7500 rpm for 10 min) 3 mL of solution and measuring the absorbance at 554 nm with a single-beam spectrophotometer (UV/Vis Hach Lange, DR 3900, Hach, Loveland, CO, USA). The photocatalytic activity was quantified as the photodegradation rate constant of catalyst k (min^−1^). The photodegradation of RhB in presence of a catalyst can be considered a pseudo-first-order reaction and can be described by Equation (1):

ln(C_0_)/(C) = kt (1)

According to the Lambert–Beer law, the absorbance is proportional to the RhB concentration, so ln (C_0_/C) is calculated by measuring the initial concentration (C_0_) and absorbance (A_0_) and after a certain irradiation time t (A_t_). The value of k was assessed by plotting ln (C_0_/C) versus time (t). The conversion, calculated at t = 120 min, indicates the ratio between the amount of reagent consumed and the amount of reagent initially present in the reaction environment; this was determined by Formula (2):

Conversion (%) = (A_0_ − A_t_)/A_0_ × 100 (2)

#### 3.4.2. Sorption Tests

The prepared samples, in powder form, were dispersed in water and kept in contact with a solution of CuCl_2_ (10 mg L^−1^) at room temperature. The tests were performed in the presence of 2.5 g L^−1^ of adsorbent samples under stirring and at a working pH of 4.5. To quantify the sorption, after keeping the samples in contact with Cu^2+^, 8 mL were centrifuged at 4500 rpm for 40 min by ultrafiltering the sample with centrifugal filter units (Polyethersulfone, Amicon filter 5 KDa, Millipore, Burlington, MA, USA). In this way, we separated the powder samples from the solution and quantified them via inductively coupled plasma atomic emission spectroscopy coupled with a OneNeb nebulizer (ICP-OES 5100—vertical dual view apparatus—Agilent Technologies, Santa Clara, CA, USA); the non-absorbed Cu^2+^ remained part of the solution despite the increase in time (1 and 24 h). The analyses were performed in radial viewing mode, and calibration curves were obtained with 0.1, 1.0, 10.0, and 100.0 mg L^−1^ standards for the element. Nitric acid was added to standards and diluted samples (1:10 *v*/*v*). The calibration curve was evaluated and showed a good correlation coefficient (R^2^) above 0.99. Results from ICP-OES were reported as the average of three independent measurements with relative standard deviation.

#### 3.4.3. Antibacterial Tests

Biocidal action was tested on TiO_2_@LP_S nanosols, obtained via sol–gel synthesis, which was deposited on polyester textile substrates via the dip-pad curing method. The textile was washed in an ultrasonic bath for 15 min in water and dried in an oven at 100 °C. Then, the washed textile was dipped in the TiO_2_@LP_S nanosols for 3 min, squeezed in two rolls to eliminate the excess of suspension (pad stage), dried in a stove at 80 °C, and finally cured at 120 °C for 10 min to fix the NPs to the fabric. A triple-layer impregnation was carried out, achieving the final dry add-on value (AO%; Equation (3)), which is defined as the percent amount of the finishing agent added to the fabric with respect to the initial weight of the latter; i.e.,

AO% = [(w_f_ − w_i_)/w_i_] × 100 (3)

where w_i_ and w_f_ are the weights of the fabric before and after the dip-pad curing process.

The antibacterial tests were assessed following the ASTM E2149 procedure and addition of Gram-positive bacteria *Staphylococcus aureus* (*S. aureus*) ATCC 6538. Before the tests, the TiO_2_@LP_S coated polyester textiles were pre-irradiated for 2 h 30 min under UV light (Osram ULTRA-Vitalux lamp 300 W). The test culture was incubated in a nutrient broth for 24 h and then diluted to a concentration of 1.5–3.0 × 10^5^ CFU mL^−1^ (working solution). Next, 1 g of each treated fabric was transferred to a flask containing 50 mL of the working solution. All flasks were shaken for 1 h at 190 rpm. After a series of dilutions, 1 ml of the solution was plated in nutrient agar. The inoculated plates were incubated at 37 °C for 24 h, and the surviving cells were counted. The biocidal action was expressed in percent bacteria reduction by counting the surviving cells after contact with the test specimen (A) compared to the number of bacterial cells in the working solution (B), according to Equation (4):

Reduction% = [(A − B)/B] × 100 (4)

#### 3.4.4. NO_x_ Abatement Test

The NO_x_ abatement test was conducted on TiO_2_@LP_S nanosol-coated textiles (10 × 10 cm), prepared via the dip-pad curing method (as described in the previous Section 3.4.3). The analyses were conducted under controlled conditions at a temperature of 26 ± 2 °C and a relative humidity of 44 ± 4% and under UV irradiation (Osram ULTRA Vitalux lamp 300 W) at an intensity of 50 W m^−2^. The analyses were performed by injecting a pollutant gas consisting of dry air, moist air, and NO in the measuring system in the presence of the coated textiles. Then, the concentration of the gases (NO, NO_x_, and NO_2_) was monitored with a chemiluminescence detector (Thermo, model 42i, Thermo Fisher Scientific, Waltham, MA, USA). The NO degradation reaction progress was monitored at regular times (25, 45, 65, 85, 105, 125, and 145 min). The kinetics of the degradation reaction were obtained, plotting the NO depletion (%) as a function of time of activation.

## 4. Conclusions

Hybrid systems based on TiO_2_ and lipopeptides (LP) components were successfully synthetized via two different approaches (sol–gel synthesis and heterocoagulation). Physicochemical and colloidal properties characterization highlighted two main populations with distinct behavior. At the breaking point, around TiO_2_:LP 1:1/1:2, the colloidal stability of the system abruptly decreased, with dramatic consequences for functional performances. On the contrary, the presence of LP at a low concentration improved the TiO_2_ dispersibility, increasing the availability of active surface sites. This resulted in the highest performances in terms of photocatalytic degradation of RhB in water, abatement of NO in gas, and antibacterial properties against *Staphylococcus aureus* of coated textiles. In particular, the hybrid system with TiO_2_:LP 1:1 preserved the high surface area of TiO_2_ (specific surface area around 180 m^2^/g), caused a NO_x_ depletion up to 100% in 80 min, and improved adhesion of the hybrid antibacterial coating, suggesting that it is the best hybrid design for the concurrent removal of inorganic, organic, and biological pollutants in water/soil remediation applications. The evaluation of potential synergic effects of the hybrid systems leads us to the following conclusions: (1) at low LP contents, the photocatalytic properties of TiO_2_ are preserved, and the hybrid systems can be used in advanced oxidation processes, taking advantage of the additional LP properties; (2) at high LP contents, TiO_2_ loses its photoactivity due to the LP coating quenching effect avoiding undesired oxidative effects; (3) in the combined adsorbent system, the TiO_2_ can improve the stability and density of the lipopetides, allowing easy transport and recovery from the medium. Overall, the eco-design of hybrid systems and the proven concurrent removal of inorganic, organic, and biological pollutants can be successfully exploited in environmental remediation technologies.

## Figures and Tables

**Table 1:** Data from colloidal characterization of TiO_2_@LP_S samples obtained via sol–gel synthesis.

Sample	d_DLS_ (nm)	Zeta-pot_ELS_ (mV)	pH_iep_
LP	nd *	−38 ± 6	1.7
TiO_2_@TX	64 ± 2	+39 ± 7	6.2
TiO_2_@LP_1:0.1_S	77 ± 4	+33 ± 6	6.1
TiO_2_@LP_1:0.5_S	215 ± 15	+28 ± 5	6.0
TiO_2_@LP_1:1_S	720 ± 143	+24 ± 4	6.0
TiO_2_@LP_1:2_S	870 ± 84	−7 ± 2	1.7
TiO_2_@LP_1:6_S	1000 ± 25	−40 ± 8	2.1
TiO_2_@LP_1:8_S	1020 ± 178	−43 ± 5	1.7

nd *, not determined.

**Table 2:** Data from colloidal characterization of TiO_2_/LP_E samples obtained via heterocoagulation.

Sample	d_DLS_ (nm)	Zeta-pot_ELS_ (mV)	pH_iep_
LP	nd *	−38 ± 6	1.7
TiO_2_@TX	64 ± 2	+39 ± 7	6.2
TiO_2_/LP_1:1_E	1100 ± 200	−16 ± 4	3.4
TiO_2_/LP_1:6_E	216 ± 5	−31 ± 58	1.7
TiO_2_/LP_1:8_E	243 ± 2	−41 ± 5	1.5

nd *, not determined.

**Table 3:** Conversion (%) and kinetic constant (min^−1^) data obtained via photocatalytic tests of TiO_2_@LP_S samples synthesized via the sol–gel method.

Sample	Conversion (%)	k (min^−1^)
TiO_2_@TX (ref.)	99	9.5 × 10^−2^
TiO_2_@LP_1:0.1_S	99	8.5 × 10^−2^
TiO_2_@LP_1:0.5_S	90	4.0 × 10^−2^
TiO_2_@LP_1:1_S	87	2.3 × 10^−2^
TiO_2_@LP_1:2_S	47	0.6 × 10^−2^
TiO_2_@LP_1:6_S	16	0.5 × 10^−2^
TiO_2_@LP_1:8_S	12	0.1 × 10^−2^

**Table 4:** Conversion (%) and kinetic constant (min^−1^) obtained via photocatalytic tests of TiO_2_/LP_E samples prepared via heterocoagulation.

Sample	Conversion (%)	k (min^−1^)
TiO_2_@TX (ref.)	99	9.5 × 10^−2^
TiO_2_/LP_1:1_E	18	0.5 × 10^−2^
TiO_2_/LP_1:6_E	5	0.6 × 10^−3^
TiO_2_/LP_1:8_E	5	0.6 × 10^−3^

**Table 5:** Results of Cu^2+^ sorption (mgCu^2+^/g_sample_) tests performed on representative samples.

Sample	Cu^2+^ Sorption (mg Cu^2+^/g_sample_)	
	1 h	24 h
LP	2.53	2.53
TiO_2_@TX (ref.)	1.36	1.39
TiO_2_@LP_1:0.1_S	1.16	1.28
TiO_2_@LP_1:1_S	1.28	1.23
TiO_2_@LP_1:8_S	2.53	2.53
TiO_2_/LP_1:1_E	1.18	1.35
TiO_2_/LP_1:8_E	2.53	2.53

**Table 6:** Results of antibacterial tests performed on representative samples.

Sample	Add-on (%)	Bacterial Reduction (%)
LP	1.7	40
TiO_2_@TX (ref.)	3.1	72
TiO_2_@LP_1:0.1_S	3.9	89
TiO_2_@LP_1:1_S	3.9	85
TiO_2_@LP_1:8_S	5.3	77

## Data Availability

The data presented in this study are available on request from the corresponding authors. The data are not publicly available due to privacy restrictions.

## Supplementary Materials

The following supporting information can be downloaded at: https://www.mdpi.com/article/10.3390/molecules28041863/s1, Table S1: Sample codes and TiO_2_:LP weight ratios of nanosols obtained via the sol–gel synthesis process and relative powders obtained via the SFD process; Table S2: Sample codes and TiO_2_:LP weight ratios of nanosols obtained via the heterocoagulation process and relative powders obtained via the SFD process; Table S3: Adsorption properties derived by UV-Vis analysis; Figure S1: Particle size distribution of TiO_2_@LP samples obtained via the sol–gel synthesis method; Figure S2: SAED patterns of the (**a**) TiO_2_@LP_S_1:0.1 and (**b**) TiO_2_@LP_S_1:1 samples; Figure S3: (**a**) Particle size distribution and (**b**) Zeta potential as a function of pH curves of TiO_2_/LP_E samples obtained via the heterocoagulation process; Figure S4: (**a**) Trends of A/A_0_ and (**b**) conversion (%) over time for TiO_2_@LP_S samples obtained via the sol–gel synthesis method; Figure S5: (**a**) Trends of A/A_0_ and (**b**) conversion (%) over time for TiO_2_/LP_E samples obtained via the heterocoagulation process; Figure S6: Scheme of (**a**) sol–gel processes using Triton X (TX) and mixture of lipopeptides (LP) as a surfactant and (**b**) the heterocoagulation process; Figure S7: Schematization of the spray-freeze-drying process; Figure S8: Diffuse reflectance over different wavelengths of TiO_2_@LP_S samples.

## Sample Availability

Samples of the compounds TiO_2_@LP and TiO_2_/LP are available from the authors.
