# Correction: Ortelli et al. Design of TiO_2_-Based Hybrid Systems with Multifunctional Properties. *Molecules* 2023, *28*, 1863

**DOI:** 10.3390/molecules29194647

**Published:** 2024-09-30

**Authors:** Simona Ortelli, Maurizio Vespignani, Ilaria Zanoni, Magda Blosi, Claudia Vineis, Andreana Piancastelli, Giovanni Baldi, Valentina Dami, Stefania Albonetti, Anna Luisa Costa

**Affiliations:** 1 CNR-ISSMC (Former ISTEC), National Research Council of Italy-Institute of Science, Technology and Sustainability for Ceramics, Via Granarolo 64, 48018 Faenza, Italyanna.costa@issmc.cnr.it (A.L.C.); 2 Department of Industrial Chemistry “Toso Montanari”, Bologna University, Viale Risorgimento 4, 40136 Bologna, Italy; 3 CNR-STIIMA, Institute of Intelligent Industrial Technologies and Systems for Advanced Manufacturing–Italian National Research Council, Corso Pella 16, 13900 Biella, Italy; claudia.vineis@stiima.cnr.it; 4 Ce.Ri.Col, Colorobbia Consulting S.R.L., 50059 Sovigliana-Vinci, Italy

There was an error in the original publication [1] regarding the biosurfactant used in this study, which we called ‘Sodium-Surfactin’ (SS). It is more accurately a crude mixture of lipopeptides (LP) derived from bacterial fermentation. This correction is made to avoid potential conflicts of interest in future publications using ‘Surfactin’, a commercial biosurfactant specifically developed for use in cosmetic applications. This is different from the biosurfactant used in this study in the optimization, purification, and characterization processes.

When the specification of the hybrid system is required, ‘Surfactin’ and ‘SS’ are substituted with ‘LipoPeptide’ and ‘LP’, respectively.

## Title Change

The word ‘Surfactin’ in the title is now substituted with ‘Based’.

## Text Correction

In addition to systematic substitution carried out across the text, the authors added the following paragraph to the Introduction, placing it after the third paragraph:

Lipopeptides are a class of biosurfactants that have been widely studied and utilized for various biomedical and environmental applications due to their diverse properties, including antimicrobial, antiadhesive, antitumor, and bioremediation activities [6–12]. Lipopeptides possess surfactant properties due to their amphiphilic nature, having both hydrophilic (peptide) and hydrophobic (lipid) components. This allows them to interact with cell membranes, disrupting their structures and functions. As a result, lipopeptides can exhibit potent antimicrobial effects against a wide range of pathogens, including bacteria, fungi, and even some viruses [13,14].

The authors rephrased the fourth paragraph of the Introduction with the following text:

Lipopeptides also exhibits good stabilizing properties used in the sol–gel synthesis of metal nanoparticles [15–19]. Thus, we decided to exploit the coupling between TiO_2_ NPs and a mixture of lipopeptides (LP), to investigate the physicochemical identity of the hybrid phase and the possible synergetic, antagonist, or independent effects in terms of functionality [20,21].

## Errors in Figure/Table Legends and Figures/Tables

The legends and/or texts of Scheme 1, Figures 1, 2, 3, 4, 5, 6, S1, S2, S3, S4, S5, S6 and S8 and Tables 1, 2, 3, 4, 5, 6, S1, S2, and S3 have been corrected. All uses of the abbreviation ‘SS’ (‘Sodium-Surfactin’) have been substituted with ‘LP’ (crude lipopeptide mixture). The corrected Figures and Tables appear below.

**Scheme 1:**
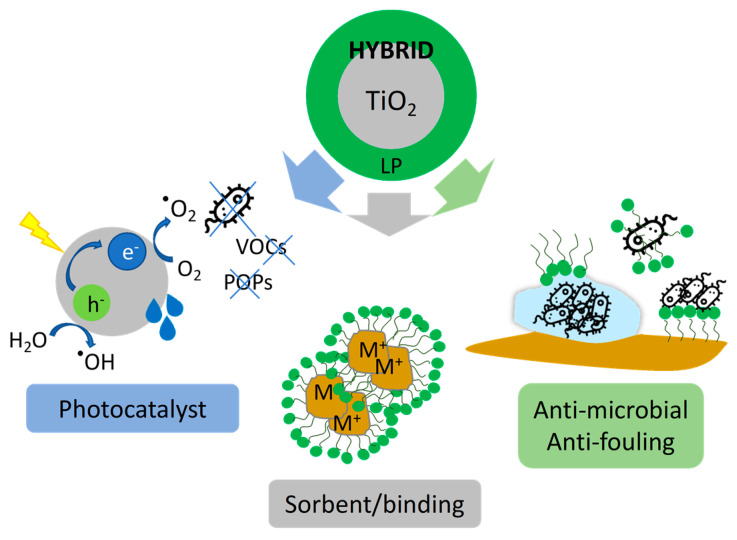
Multifunctional platform designed for the removal of water/soil pollutants.

**Figure 1:**
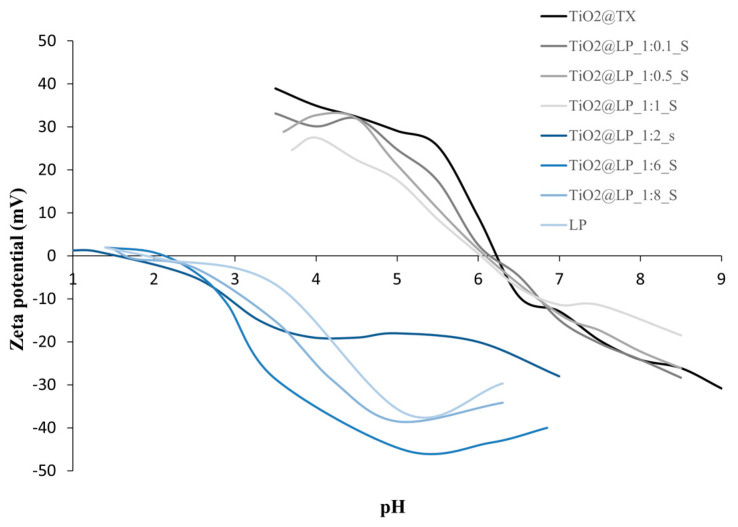
Zeta potential as a function of pH curves for TiO_2_@LP_S samples obtained via sol–gel synthesis.

**Figure 2:**
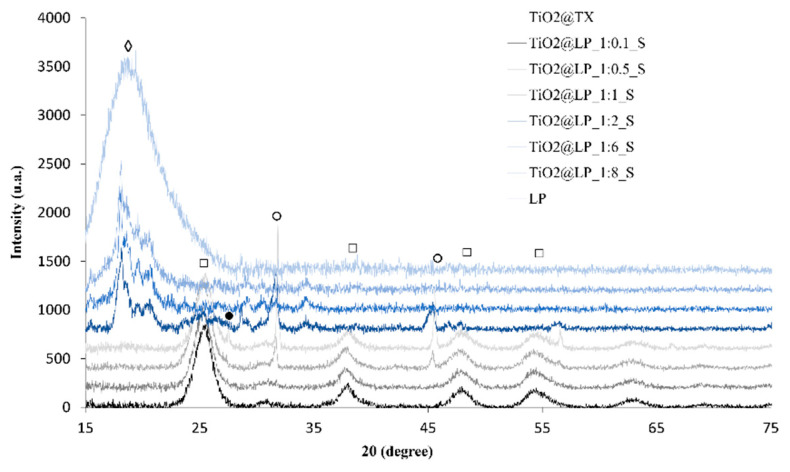
XRD diffractograms of TiO_2_@LP_S_SFD samples (◊, LP; □, anatase; ●, brookite; ○, sodium chloride).

**Figure 3:**
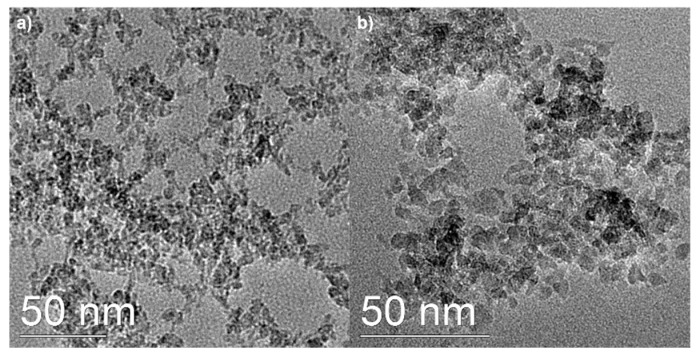
TEM images of (**a**) TiO_2_@LP_S_1:0.1 and (**b**) TiO_2_@LP_S_1:1 samples.

**Figure 4:**
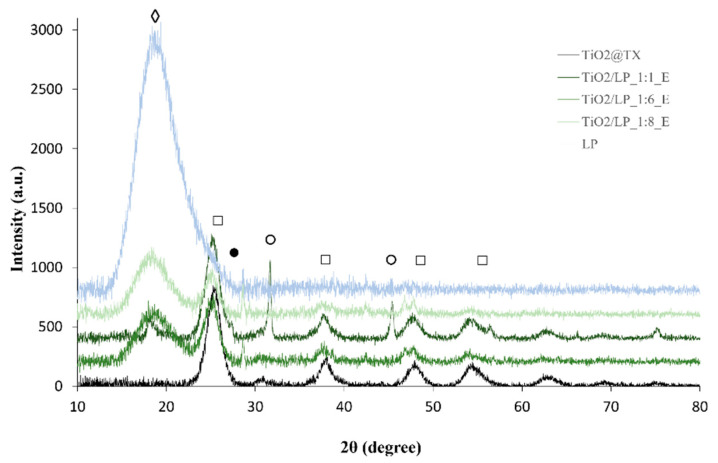
XRD diffractograms of TiO_2_/LP_E_SFD samples (◊, LP; □, anatase; ●, brookite; ○, sodium chloride).

**Figure 5:**
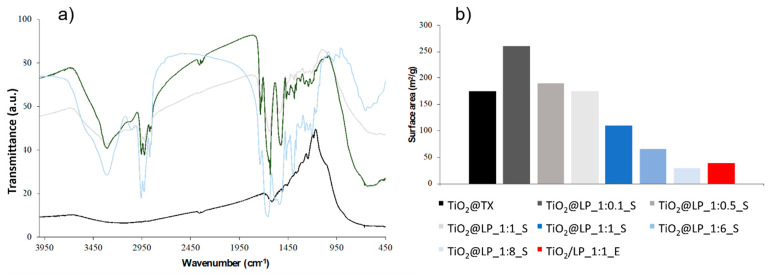
(**a**) FTIR spectra of TiO_2_@TX (black), LP (light blue), TiO_2_@LP_1:1_S (light gray), and TiO_2_/LP_1:1_E (dark green) samples and (**b**) specific surface area data (m^2^/g).

**Figure 6:**
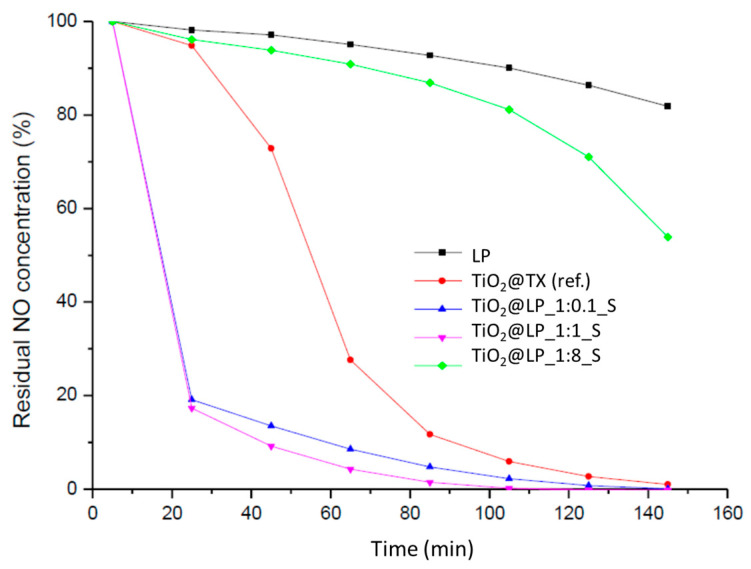
NO depletion trend as a function of UV light time of irradiation.

**Table 1:** Data from colloidal characterization of TiO_2_@LP_S samples obtained via sol–gel synthesis.

Sample	d_DLS_ (nm)	Zeta-pot_ELS_ (mV)	pH_iep_
LP	nd *	−38 ± 6	1.7
TiO_2_@TX	64 ± 2	+39 ± 7	6.2
TiO_2_@LP_1:0.1_S	77 ± 4	+33 ± 6	6.1
TiO_2_@LP_1:0.5_S	215 ± 15	+28 ± 5	6.0
TiO_2_@LP_1:1_S	720 ± 143	+24 ± 4	6.0
TiO_2_@LP_1:2_S	870 ± 84	−7 ± 2	1.7
TiO_2_@LP_1:6_S	1000 ± 25	−40 ± 8	2.1
TiO_2_@LP_1:8_S	1020 ± 178	−43 ± 5	1.7

nd *, not determined.

**Table 2:** Data from colloidal characterization of TiO_2_/LP_E samples obtained via heterocoagulation.

Sample	d_DLS_ (nm)	Zeta-pot_ELS_ (mV)	pH_iep_
LP	nd *	−38 ± 6	1.7
TiO_2_@TX	64 ± 2	+39 ± 7	6.2
TiO_2_/LP_1:1_E	1100 ± 200	−16 ± 4	3.4
TiO_2_/LP_1:6_E	216 ± 5	−31 ± 58	1.7
TiO_2_/LP_1:8_E	243 ± 2	−41 ± 5	1.5

nd *, not determined.

**Table 3:** Conversion (%) and kinetic constant (min^−1^) data obtained via photocatalytic tests of TiO_2_@LP_S samples synthesized via the sol–gel method.

Sample	Conversion (%)	k (min^−1^)
TiO_2_@TX (ref.)	99	9.5 × 10^−2^
TiO_2_@LP_1:0.1_S	99	8.5 × 10^−2^
TiO_2_@LP_1:0.5_S	90	4.0 × 10^−2^
TiO_2_@LP_1:1_S	87	2.3 × 10^−2^
TiO_2_@LP_1:2_S	47	0.6 × 10^−2^
TiO_2_@LP_1:6_S	16	0.5 × 10^−2^
TiO_2_@LP_1:8_S	12	0.1 × 10^−2^

**Table 4:** Conversion (%) and kinetic constant (min^−1^) obtained via photocatalytic tests of TiO_2_/LP_E samples prepared via heterocoagulation.

Sample	Conversion (%)	k (min^−1^)
TiO_2_@TX (ref.)	99	9.5 × 10^−2^
TiO_2_/LP_1:1_E	18	0.5 × 10^−2^
TiO_2_/LP_1:6_E	5	0.6 × 10^−3^
TiO_2_/LP_1:8_E	5	0.6 × 10^−3^

**Table 5:** Results of Cu^2+^ sorption (mgCu^2+^/g_sample_) tests performed on representative samples.

Sample	Cu^2+^ Sorption (mg Cu^2+^/g_sample_)	
	1 h	24 h
LP	2.53	2.53
TiO_2_@TX (ref.)	1.36	1.39
TiO_2_@LP_1:0.1_S	1.16	1.28
TiO_2_@LP_1:1_S	1.28	1.23
TiO_2_@LP_1:8_S	2.53	2.53
TiO_2_/LP_1:1_E	1.18	1.35
TiO_2_/LP_1:8_E	2.53	2.53

**Table 6:** Results of antibacterial tests performed on representative samples.

Sample	Add-on (%)	Bacterial Reduction (%)
LP	1.7	40
TiO_2_@TX (ref.)	3.1	72
TiO_2_@LP_1:0.1_S	3.9	89
TiO_2_@LP_1:1_S	3.9	85
TiO_2_@LP_1:8_S	5.3	77

**Figure S1:**
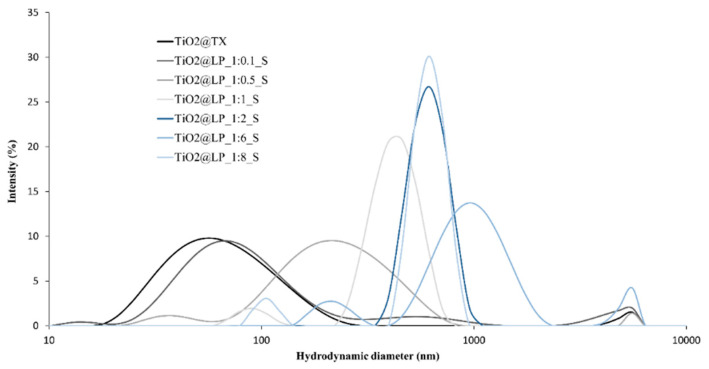
Particle size distribution of TiO_2_@LP samples obtained via the sol–gel synthesis method.

**Figure S2:**
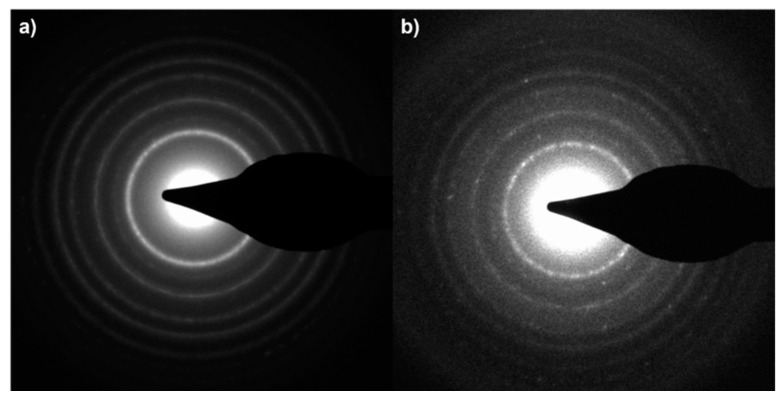
SAED patterns of the (**a**) TiO_2_@LP_S_1:0.1 and (**b**) TiO_2_@LP_S_1:1 samples.

**Figure S3:**
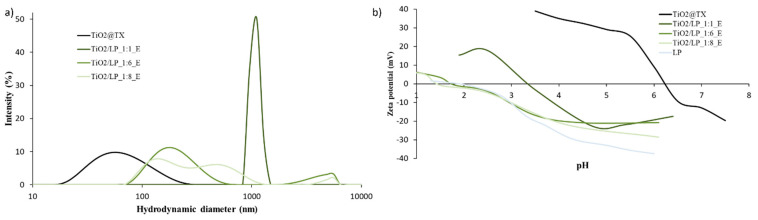
(**a**) Particle size distribution and (**b**) Zeta potential as a function of pH curves of TiO_2_/LP_E samples obtained via the heterocoagulation process.

**Figure S4:**
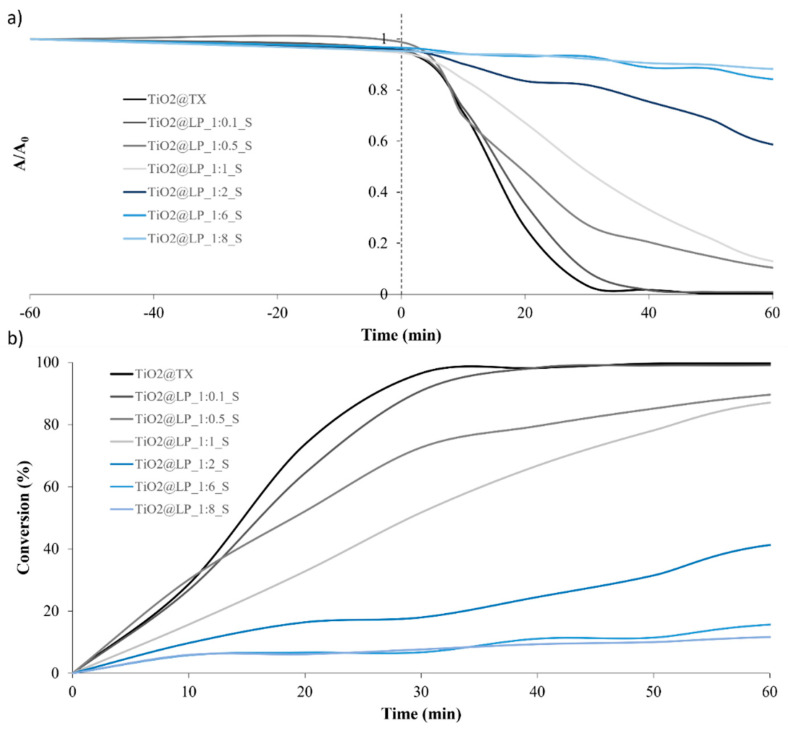
(**a**) Trends of A/A_0_ and (**b**) conversion (%) over time for TiO_2_@LP_S samples obtained via the sol–gel synthesis method.

**Figure S5:**
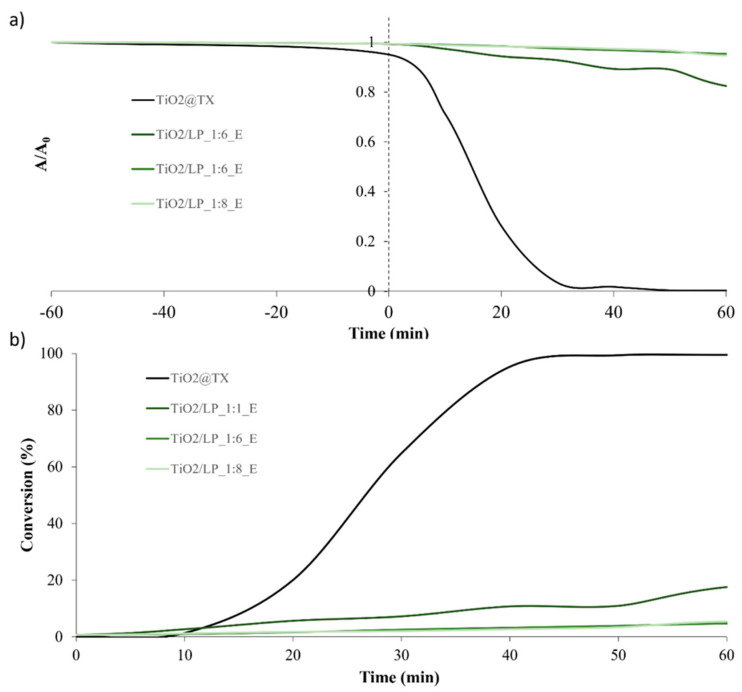
(**a**) Trends of A/A_0_ and (**b**) conversion (%) over time for TiO_2_/LP_E samples obtained via the heterocoagulation process.

**Figure S6:**
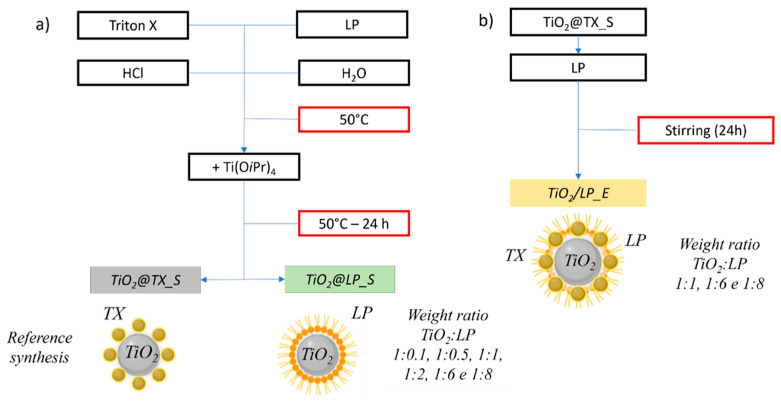
Scheme of (**a**) sol–gel processes using Triton X (TX) and mixture of lipopeptides (LP) as a surfactant and (**b**) the heterocoagulation process.

**Figure S8:**
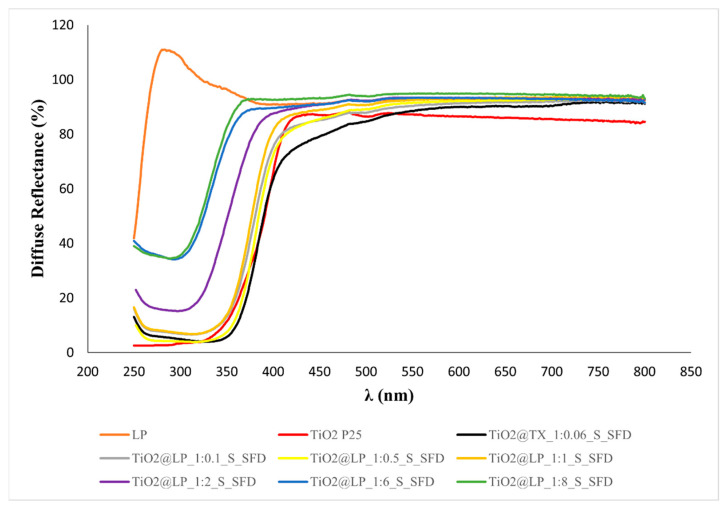
Diffuse reflectance over different wavelengths of TiO_2_@LP_S samples.

**Table S1:** Sample codes and TiO_2_:LP weight ratios of nanosols obtained via the sol–gel synthesis process and relative powders obtained via the SFD process.

Sample Code		TiO_2_:LP Weight Ratio
Nanosol	Powder	
**TiO_2_@LP_1:0.1_S**	TiO_2_@LP_1:0.1_S_SFD	10.0
**TiO_2_@LP_1:0.5_S**	TiO_2_@LP_1:0.5_S_SFD	2.0
**TiO_2_@LP_1:1_S**	TiO_2_@LP_1:1_S_SFD	1.0
**TiO_2_@LP_1:2_S**	TiO_2_@LP_1:2_S_SFD	0.5
**TiO_2_@LP_1:6_S**	TiO_2_@LP_1:6_S_SFD	0.17
**TiO_2_@LP_1:8_S**	TiO_2_@LP_1:8_S_SFD	0.13
**TiO_2_@TX_S**	TiO_2_@TX_S_SFD	16.7 *

* TiO_2_:Triton X weight ratio.

**Table S2:** Sample codes and TiO_2_:LP weight ratios of nanosols obtained via the heterocoagulation process and relative powders obtained via the SFD process.

Sample Code		TiO_2_:LP Weight Ratio
Nanosol Sample	Powder Sample	
**TiO_2_/LP_1:1_E**	TiO_2_/LP_1:1_E_SFD	1.0
**TiO_2_/LP_1:6_E**	TiO_2_/LP_1:6_E_SFD	0.17
**TiO_2_/LP_1:8_E**	TiO_2_/LP_1:8_E_SFD	0.13

The TiO_2_ used to produce the heterocoagulated samples is TiO_2_@TX_S of Table S1 (containing around 6 wt.% of Triton X).

**Table S3:** Adsorption properties derived by UV-Vis analysis.

Powder Sample Code	Absorption Range (nm)	Band Gap Energy (eV)
**TiO_2_@TX_SFD**	350–450	3.14
TiO_2_ P25 *	350–420	3.19
**TiO_2_@LP_1:0.1_S_SFD**	350–420	3.17
**TiO_2_@LP_1:0.5_S_SFD**	350–420	3.18
**TiO_2_@LP_1:1_S_SFD**	350–420	3.18
**TiO_2_@LP_1:2_S_SFD**	300–380	3.33
**TiO_2_@LP_1:6_S_SFD**	300–360	3.41
**TiO_2_@LP_1:8_S_SFD**	300–360	3.41

* TiO_2_ P25 (commercial powder sample from Degussa-Evonik).

## Revised References

Previous references focused exclusively on Surfactin. Due to the proposed correction (substitution of Surfactin with a mixture of lipopeptides), it was necessary to revise references specifically addressing lipopeptides to strengthen the rationale behind this study.

References 7 and 10 from the original publication [1] have now been removed. Also, the following citations have now been inserted as references 6, 10, 12, 15, and 16 into the Introduction. With this correction, the order of some references has been adjusted accordingly.

6. Meena, K.R.; Kanwar, S.S. Lipopeptides as Antifungal and Antibacterial Agents: Applications in Food Safety and Therapeutics. *BioMed Res. Int.* **2015**, *2015*, 473050. https://doi.org/10.1155/2015/473050.
10. Inès, M.; Dhouha, G. Lipopeptide Surfactants: Production, Recovery and Pore Forming Capacity. *Peptides* **2015**, *71*, 100–112. https://doi.org/10.1016/j.peptides.2015.07.006.
12. Kourmentza, C.; Freitas, F.; Alves, V.; Reis, M.A.M. Microbial conversion of waste and surplus materials into high-value added products: the case of biosurfactants. In *Microbial Applications*; Kalia, V., Kumar, P., Eds.; Springer: Cham, Switzerland, 2017; Volume 1, pp. 29–77. https://doi.org/10.1007/978-3-319-52666-9_2.
15. Sharma, R.K.; Dey, G.; Banerjee, P.; Maity, J.P.; Lu, C.M.; Siddique, J.A.; Wang, S.C.; Chatterjee, N.; Das, K.; Chen, C.Y. New Aspects of Lipopeptide-Incorporated Nanoparticle Synthesis and Recent Advancements in Biomedical and Environmental Sciences: A Review. *J. Mater. Chem. B* **2023**, *11*, 10–32. https://doi.org/10.1039/D2TB01564A.
16. Christopher, F.C.; Ponnusamy, S.K.; Ganesan, J.J.; Ramamurthy, R. Investigating the Prospects of Bacterial Biosurfactants for Metal Nanoparticle Synthesis—A Comprehensive Review. *IET Nanobiotechnol.* **2019**, *13*, 243. https://doi.org/10.1049/iet-nbt.2018.5184.

## Missing Conflict of Interest

In the original publication [1], the Conflicts of Interest statement of authors Giovanni Baldi and Valentina Dami were not included. The updated Conflict of Interest has been added as follows:

Giovanni Baldi and Valentina Dami was employed by the company Ce.Ri.Col, Colorobbia Consulting S.R.L. The remaining authors declare that the research was conducted in the absence of any commercial or financial relationships that could be construed as a potential conflict of interest.

## Author’s Information

This article has been republished with a minor update to the correspondence contact information.

The authors state that the scientific conclusions are unaffected. This correction was approved by the Academic Editor. The original publication has also been updated.
