# Supplementary material for: Design of TiO2-Based Hybrid Systems with Multifunctional Properties
Source: Molecules. 2023 Feb 16;28(4):1863. doi: 10.3390/molecules28041863 (PMC9967613; doi:10.3390/molecules28041863)
Supplement: Supplementary file 1 [file molecules-28-01863-s001.zip › molecules-3139960-supplementary.pdf]

Design of TiO_2-Based Hybrid Systems with Multifunctional Properties

Simona Ortelli , Maurizio Vespignani, Ilaria Zanoni, Magda Blosi, Claudia Vineis, Andreana Piancastelli, Giovanni Baldi, Valentina Dami, Stefania Albonetti and Anna Luisa Costa

Supplementary Materials

Table S1. Sample codes and TiO_2:LP weight ratios of nanosols obtained via the sol–gel synthesis process and relative powders obtained via the SFD process.

Sample Code		TiO_2:LP Weight Ratio
Nanosol	Powder	
TiO_2@LP_1:0.1_S	TiO_2@LP_1:0.1_S_SFD	10.0
TiO_2@LP_1:0.5_S	TiO_2@LP_1:0.5_S_SFD	2.0
TiO_2@LP_1:1_S	TiO_2@LP_1:1_S_SFD	1.0
TiO_2@LP_1:2_S	TiO_2@LP_1:2_S_SFD	0.5
TiO_2@LP_1:6_S	TiO_2@LP_1:6_S_SFD	0.17
TiO_2@LP_1:8_S	TiO_2@LP_1:8_S_SFD	0.13
TiO_2@TX_S	TiO_2@TX_S_SFD	16.7 *

* TiO_2:Triton X weight ratio.

Table S2. Sample codes and TiO_2:LP weight ratios of nanosols obtained via the heterocoagulation process and relative powders obtained via the SFD process.

Sample Code		TiO_2:LP Weight Ratio
Nanosol Sample	Powder Sample	
TiO_2/LP_1:1_E	TiO_2/LP_1:1_E_SFD	1.0
TiO_2/LP_1:6_E	TiO_2/LP_1:6_E_SFD	0.17
TiO_2/LP_1:8_E	TiO_2/LP_1:8_E_SFD	0.13

The TiO_2 used to produce the heterocoagulated samples is TiO_2@TX_S of Table S1 (containing around 6 wt.% of Triton X).

Table S3. Adsorption properties derived by UV-Vis analysis.

Powder Sample Code	Absorption Range (nm)	Band Gap Energy (eV)
**TiO_2@TX_SFD**	350–450	3.14
**TiO_2 P25 ***	350–420	3.19
**TiO_2@LP_1:0.1_S_SFD**	350–420	3.17
**TiO_2@LP_1:0.5_S_SFD**	350–420	3.18
**TiO_2@LP_1:1_S_SFD**	350–420	3.18
**TiO_2@LP_1:2_S_SFD**	300–380	3.33
**TiO_2@LP_1:6_S_SFD**	300–360	3.41
**TiO_2@LP_1:8_S_SFD**	300–360	3.41

* TiO_2 P25 (commercial powder sample from Degussa-Evonik).

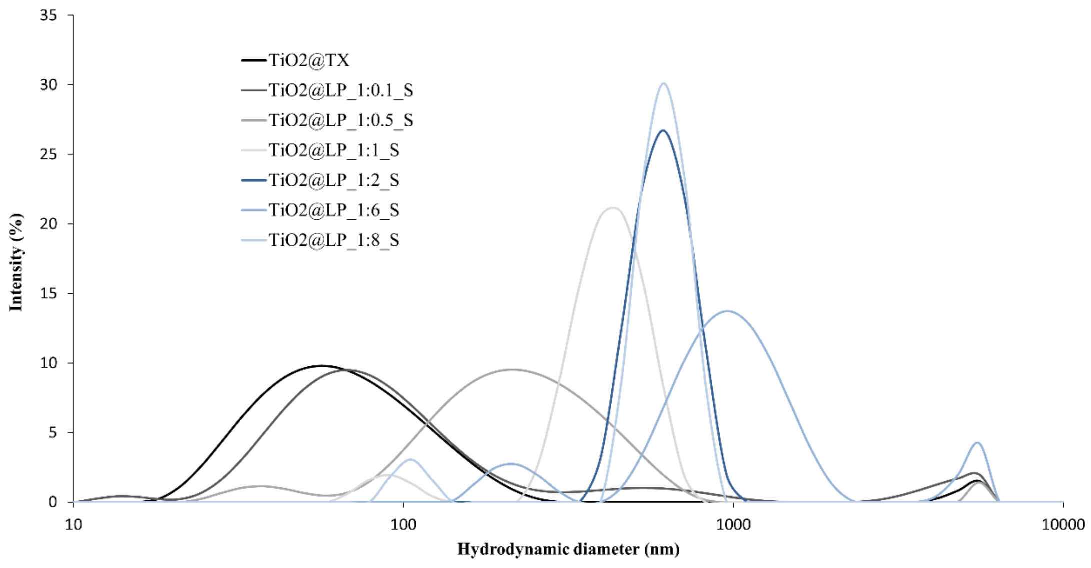

Figure S1. Particle size distribution of TiO_2@LP samples obtained via the sol–gel synthesis method.

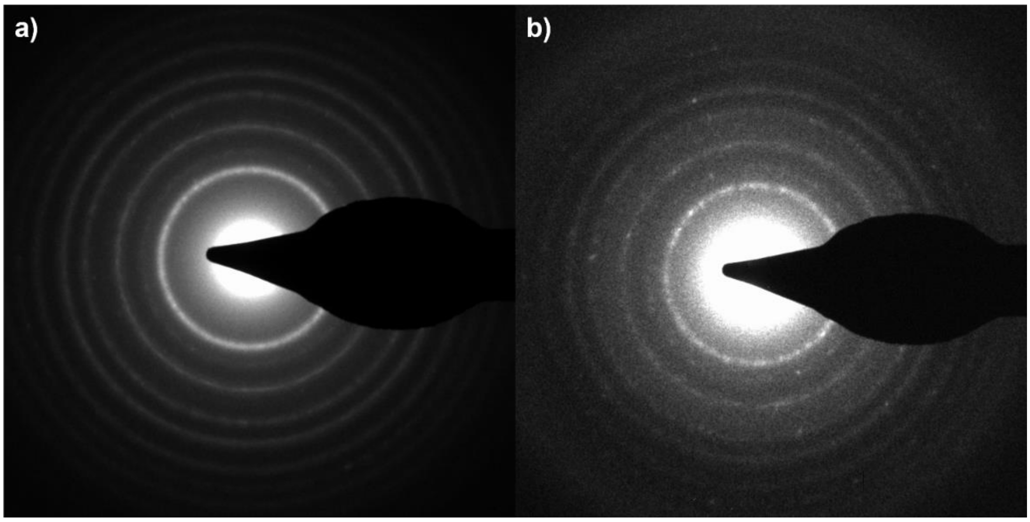

Figure S2. SAED patterns of the (**a**) TiO_2@LP_S_1:0.1 and (**b**) TiO_2@LP_S_1:1 samples.

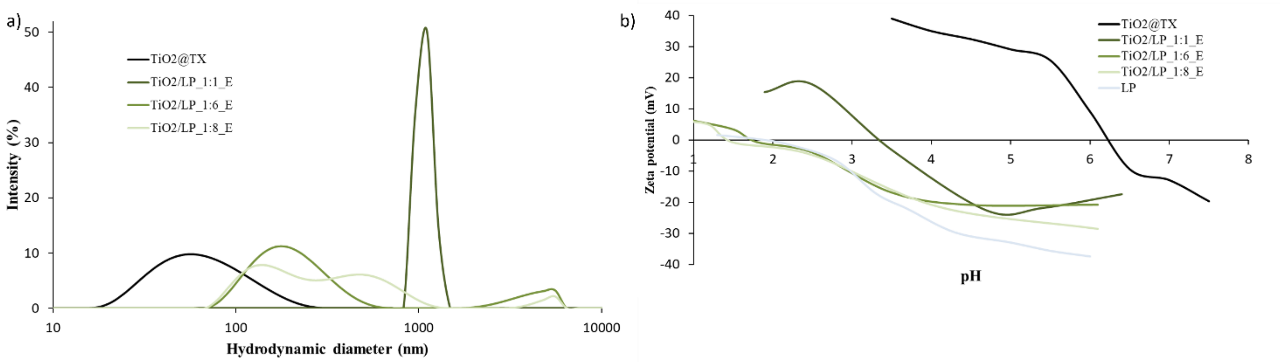

Figure S3. (**a**) Particle size distribution and (**b**) Zeta potential as a function of pH curves of TiO_2/LP_E samples obtained via the heterocoagulation process.

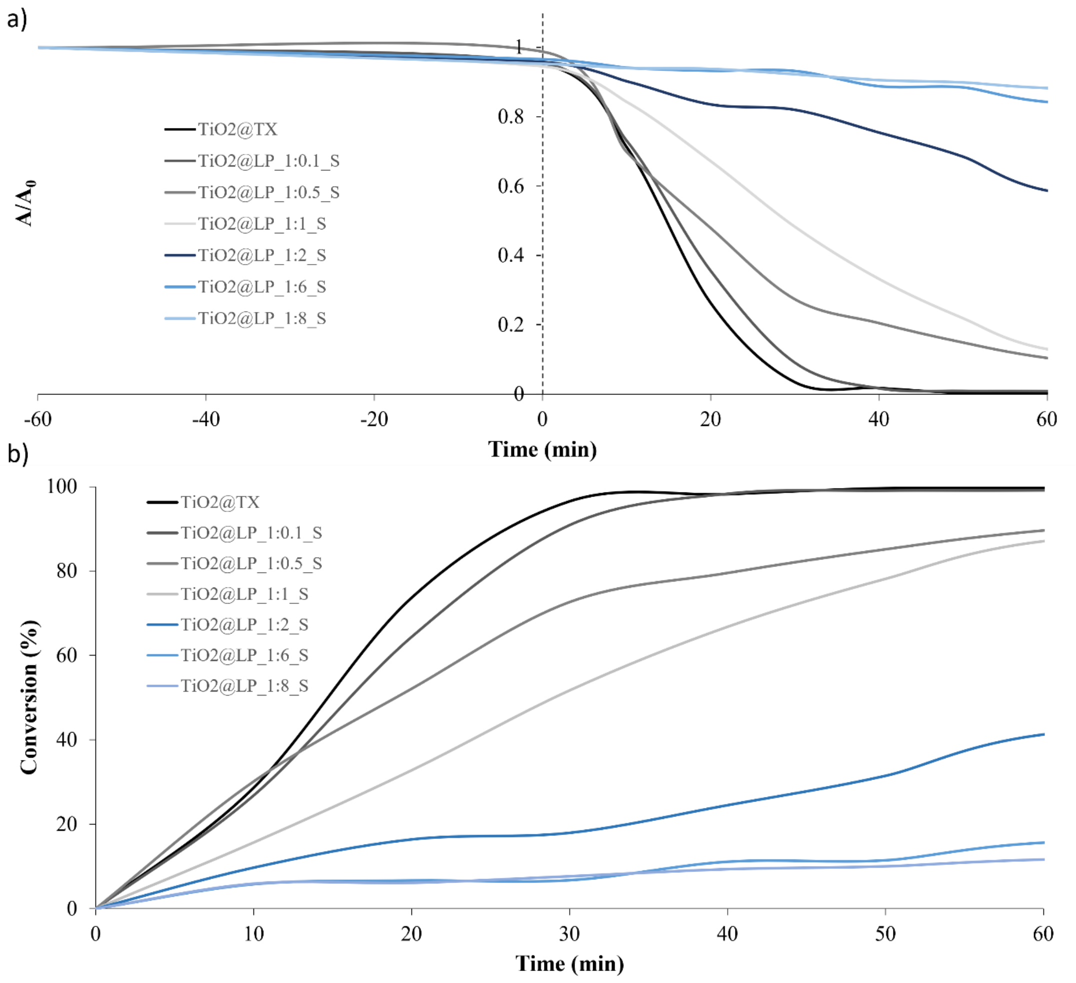

Figure S4. (**a**) Trends of A/A_0 and (**b**) conversion (%) over time for TiO_2@LP_S samples obtained via the sol–gel synthesis method.

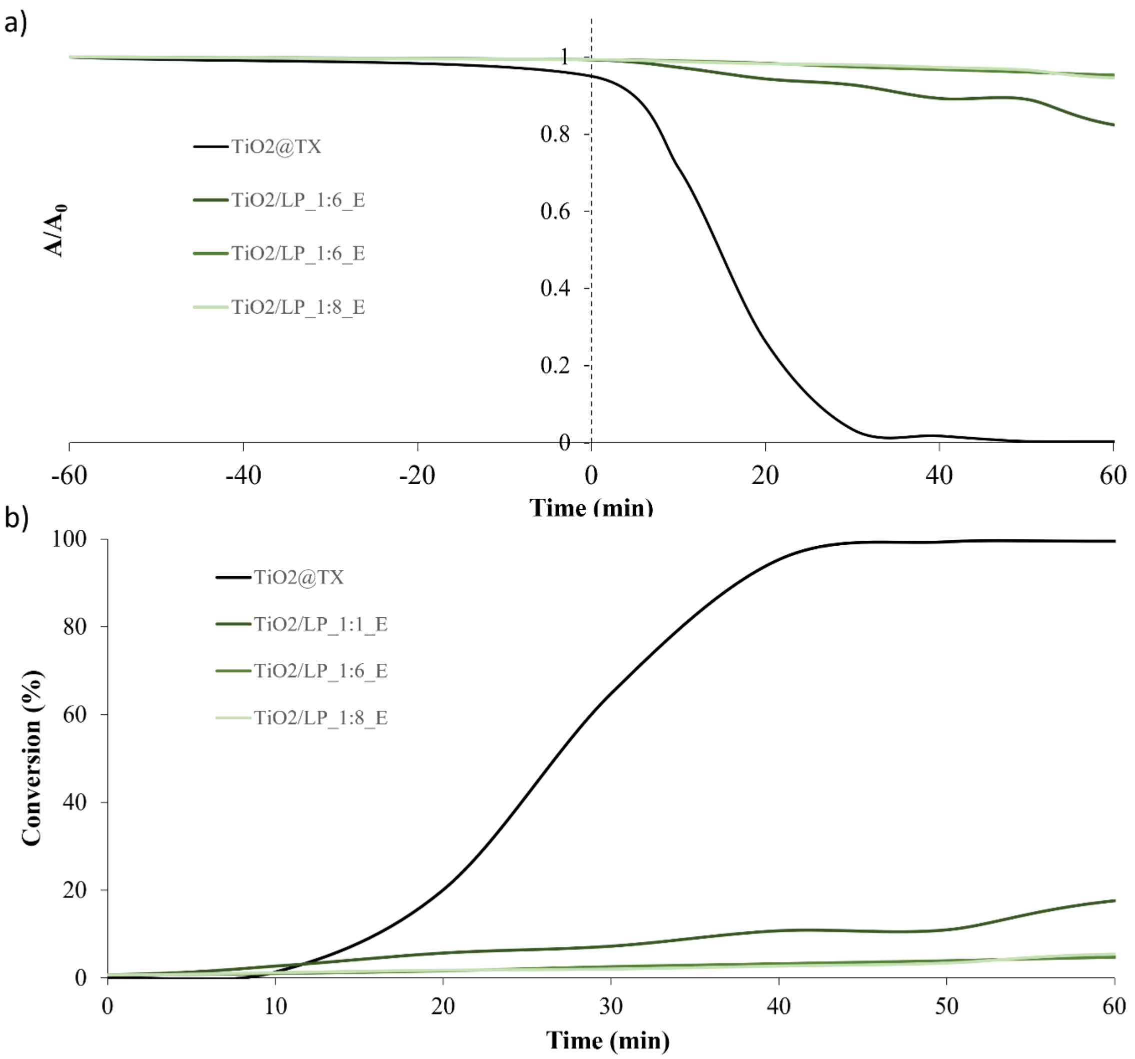

Figure S5. (**a**) Trends of A/A_0 and (**b**) conversion (%) over time for TiO_2/LP_E samples obtained via the heterocoagulation process.

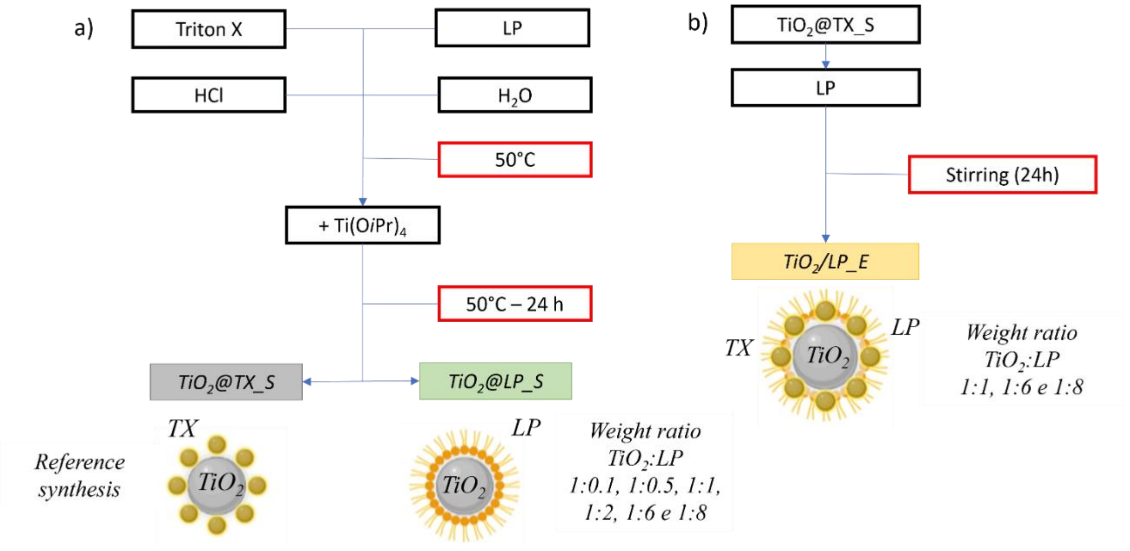

Figure S6. Scheme of (**a**) sol–gel processes using Triton X (TX) and mixture of lipopeptides (LP) as a surfactant and (**b**) the heterocoagulation process.

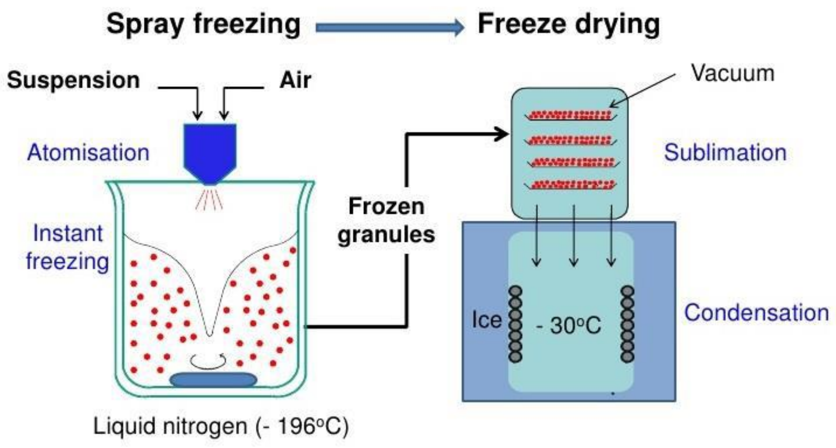

Figure S7. Schematisation of the spray-freeze-drying process.

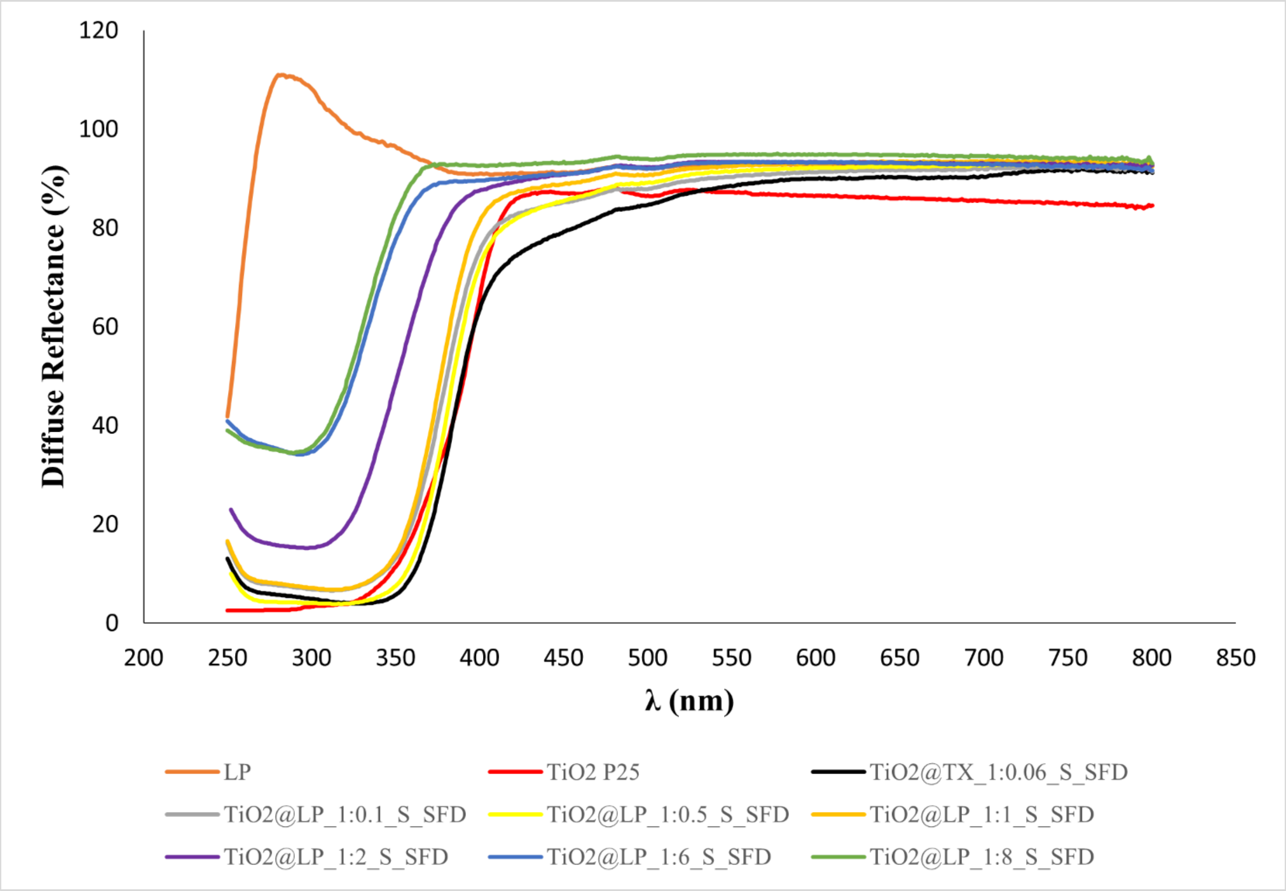

Figure S8. Diffuse reflectance over different wavelengths of TiO_2@LP_S samples.
